# Charge-Surrounded Pockets and Electrostatic Interactions with Small Ions Modulate the Activity of Retroviral Fusion Proteins

**DOI:** 10.1371/journal.ppat.1001268

**Published:** 2011-02-03

**Authors:** Daniel Lamb, Alexander W. Schüttelkopf, Daan M. F. van Aalten, David W. Brighty

**Affiliations:** 1 The Biomedical Research Institute, College of Medicine, Ninewells Hospital, The University of Dundee, Dundee, United Kingdom; 2 Division of Molecular Microbiology, College of Life Sciences, University of Dundee, Dundee, United Kingdom; Harvard Medical School, United States of America

## Abstract

Refolding of viral class-1 membrane fusion proteins from a native state to a trimer-of-hairpins structure promotes entry of viruses into cells. Here we present the structure of the bovine leukaemia virus transmembrane glycoprotein (TM) and identify a group of asparagine residues at the membrane-distal end of the trimer-of-hairpins that is strikingly conserved among divergent viruses. These asparagines are not essential for surface display of pre-fusogenic envelope. Instead, substitution of these residues dramatically disrupts membrane fusion. Our data indicate that, through electrostatic interactions with a chloride ion, the asparagine residues promote assembly and profoundly stabilize the fusion-active structures that are required for viral envelope-mediated membrane fusion. Moreover, the BLV TM structure also reveals a charge-surrounded hydrophobic pocket on the central coiled coil and interactions with basic residues that cluster around this pocket are critical to membrane fusion and form a target for peptide inhibitors of envelope function. Charge-surrounded pockets and electrostatic interactions with small ions are common among class-1 fusion proteins, suggesting that small molecules that specifically target such motifs should prevent assembly of the trimer-of-hairpins and be of value as therapeutic inhibitors of viral entry.

## Author Summary

Human T-cell leukaemia virus types-1 (HTLV-1) and bovine leukaemia virus (BLV) are divergent blood borne viruses that cause hematological malignancies in humans and cattle respectively. In common with other enveloped viruses, infection of cells by HTLV-1 and BLV is dependent on the membrane fusion properties of the viral envelope glycoproteins. Here we have solved the crystal structure of the BLV transmembrane glycoprotein, and, through a functional and comparative analysis with HTLV-1, we have identified features that are critical to fusion protein function. In particular, we demonstrate that electrostatic interactions with small ions dramatically stabilize the assembly and fusion-associated forms of the BLV TM, but are not required for the cell surface display of native pre-fusogenic envelope. Moreover, we show that charged residues that border a deep hydrophobic pocket contribute directly to appropriate folding of fusion-active envelope and are critical to membrane fusion. Importantly, the charged residues that border the pocket are key features that determine the specificity and activity of peptide inhibitors of envelope function. Our study demonstrates that charge-surrounded pockets and electrostatic interactions with small ions are significant leitmotifs of diverse class-1 fusion proteins and that these elements represent ideal targets for novel small-molecule inhibitors of viral entry.

## Introduction

Bovine Leukemia Virus (BLV) and Human T-Cell Leukemia Virus Type-1 (HTLV-1) are related deltaretroviruses that cause aggressive lymphoproliferative disorders in a small percentage of infected hosts [1], [2], [3], [4], [5], [6]. Like other enveloped viruses, retroviruses must catalyse fusion of the viral and target cell membranes to promote entry of the viral capsid into the target cell. The retroviral class I fusion protein consists of the transmembrane glycoprotein (TM) component of the envelope glycoprotein complex [7]. Envelope is displayed on the surface of the virus or infected cell as a trimer, with three surface glycoprotein (SU) subunits linked by disulphide bonds to a spike of three TM subunits [8]. Experimentally validated models suggest that SU-mediated receptor engagement induces isomerisation of the inter-subunit disulphide bonds and initiates a cascade of conformational changes that activate the fusogenic properties of TM [9], [10]. Membrane fusion is achieved by re-folding of the TM from a native non-fusogenic structure through a rod-like pre-hairpin intermediate, in which the C- and N-terminal segments are embedded in the viral and target cell membranes respectively [7], [8]. The pre-hairpin intermediate then resolves to a trimer-of-hairpins structure, which pulls the membranes together and facilitates lipid mixing and membrane fusion [7], [8], [11], [12]. For several viruses membrane fusion is sensitive to inhibition by peptides that mimic a C-terminal region of the trimer-of-hairpins [13], [14], [15], [16], [17], [18], [19]. The C-terminal fragment of the HTLV-1 trimer-of-hairpins exhibits a short α-helical motif embedded in an extended non-helical peptide structure referred to as the leash and α-helical region (LHR) [20], [21]. The LHR-based mimetics are structurally distinct from the prototypic extensively α-helical peptide inhibitors of human immunodeficiency virus but are reminiscent of the leash regions observed in influenza haemagglutinin [20], [21], [22], [23].

Importantly, amino acid residues that are required for potent inhibitory activity of the HTLV-1 and BLV peptides are not fully resolved in the available HTLV-1 TM structure, yet this information is critical to the development of therapeutically relevant peptide or low-molecular-weight inhibitors of HTLV-1 entry [17], [22]. Moreover, other class I fusion proteins have extended non-helical elements in the C-terminal region of the trimer-of-hairpins and understanding how these elements contribute to the leash in a groove mechanism of fusion protein function will have broad relevance to anti-viral therapies [23], [24]. We therefore sought to examine the structure and function of the BLV TM and to compare this information with data derived from diverse class I fusion proteins and the related HTLV-1 TM structure.

Here we show the structure of the post-fusion conformation of the BLV TM ectodomain and demonstrate that coordinated ions and a network of hydrogen-bonded water molecules make critical contributions to the assembly and stability of the trimer-of-hairpins form of the BLV TM and are essential for TM-mediated fusion. Additionally, we resolve a region of the LHR that is critical to the activity of peptide inhibitors of HTLV-1 and BLV entry. We provide evidence that basic residues in a membrane proximal helical element of the LHR interact with charged residues that surround an extended hydrophobic pocket on the coiled coil. This charge-surrounded pocket represents an attractive target for antiviral drugs. Our data indicate that coordinated ions and charge-surrounded hydrophobic pockets are functionally significant leitmotifs of class I fusion proteins.

## Results

### The structure of the BLV TM ectodomain

**Figure 1:**
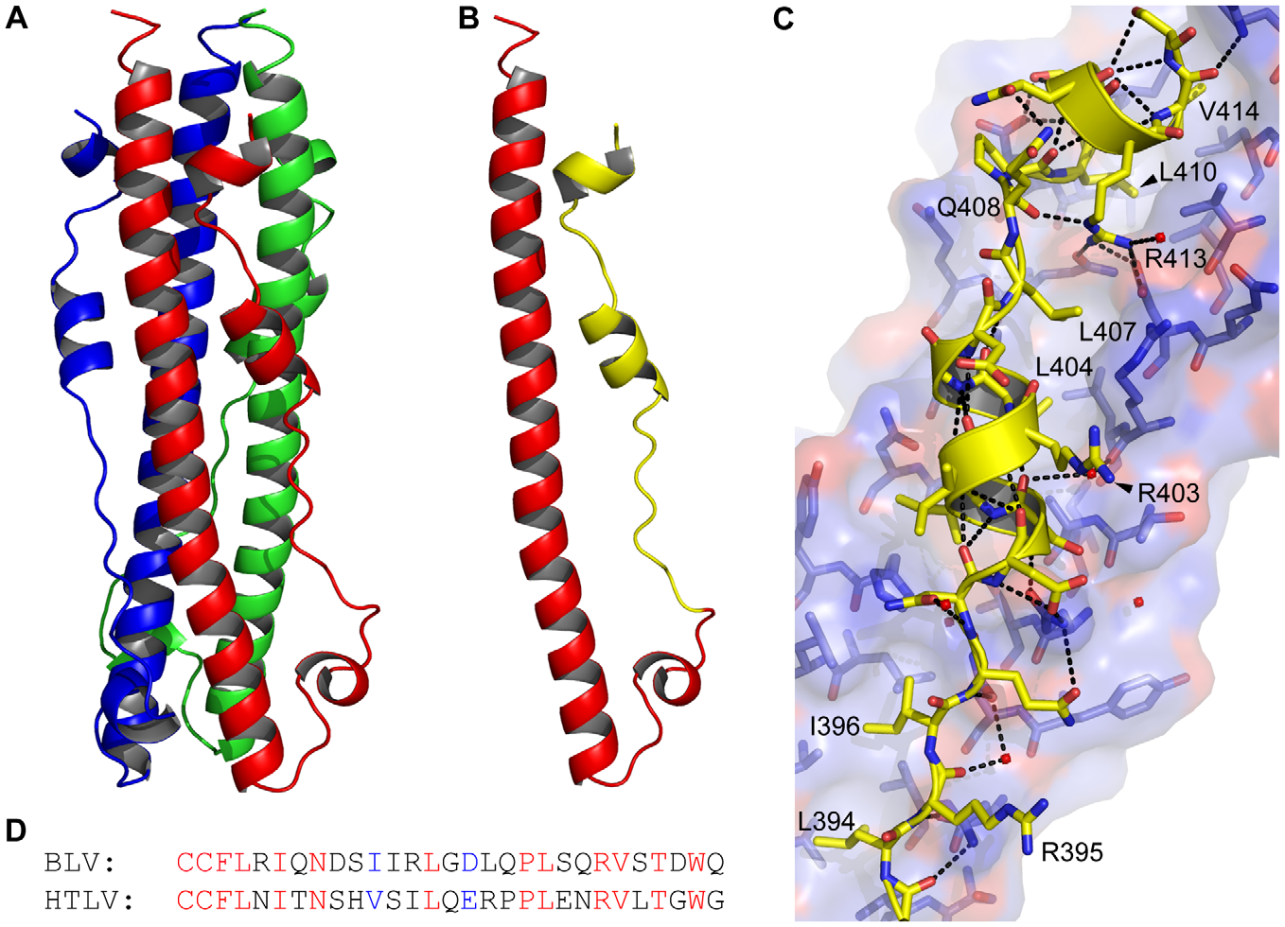
The BLV trimer-of-hairpins. (**A**) Crystal structure of the BLV trimer-of-hairpins shown as a cartoon, each monomer is differently coloured. (**B**) Cartoon representation of the BLV hairpin monomer displaying the N-terminal helical region and chain reversal regions (red) and the leash and helical regions (LHR, yellow). The membrane proximal end is at the top. (**C**) Detail of the interaction of the BLV LHR (yellow cartoon and sticks), with the coiled coil shown in blue and red. Dashed lines represent potential hydrogen bonds. Important residues in the BLV coiled coil are shown as sticks, key LHR residues are annotated. (**D**) Alignment of the BLV and HTLV-1 LHRs. Conserved residues are shown in red, semi-conserved in blue and non-conserved in black. Structural diagrams were produced using PyMol (Amino acid coordinates are BLV Cys391-Gln419; and HTLV-1 Cys400-Leu429).

To obtain crystals for structural studies, the N- and C-terminal limits of the coiled coil region of BLV TM were identified using LearnCoil VMF [25]. The TM ectodomain, including the predicted coiled coil and LHR from Ala326 to Trp418, were fused to the C-terminal end of maltose binding protein via a three-alanine spacer following the methodology of Center *et al* [26]. The soluble purified protein was crystallised and the structure solved to a resolution of 2.0 Å (Fig. 1). As anticipated, the overall fold of the BLV TM ectodomain is that of a trimer-of-hairpins, with the N-terminal α-helices twisting around each other to produce the central triple-stranded coiled coil that is characteristic of class I fusion proteins. Buried leucine and isoleucine residues within the coiled coil predominantly mediate the interactions between monomers, but strikingly there are two polar layers within the coiled coil that establish interactions with ordered water molecules and a chloride ion respectively (see below). At the base of the coiled coil the peptide backbone undergoes a 180° loop forming the chain reversal region (Fig. 1A, B), within which is a short helical segment containing the first cysteine of the conserved CX_6_CC motif [9], [10]. The predicted disulphide between Cys384 and Cys391 is reduced in the resolved structure but this does not appear to affect the overall protein fold. In support of this view, preliminary lower resolution structures obtained for crystals formed in the absence of TCEP-HCL reveal an intact disulphide (data not shown). These data indicate that the disulphide is not essential for constraining the chain reversal region in the folded protein, even though it might play a role in the folding process. Therefore, the known defects in membrane fusion associated with substitution of these cysteines are likely due to direct perturbation of the inter-subunit disulphide isomerisation step of the envelope-mediated membrane fusion process [9], [10]. After Cys391, the LHR begins with an extended non-helical leash followed by an eight-residue α-helix. This helix is followed by a three-residue linker incorporating a single proline, allowing a sharp kink in the LHR prior to the start of a second α-helix that adopts an orientation almost 90° to the first helical segment of the LHR (Fig. 1A, B, C).

### A panel of mutants in BLV envelope are processed and expressed identically in vivo

**Figure 2:**
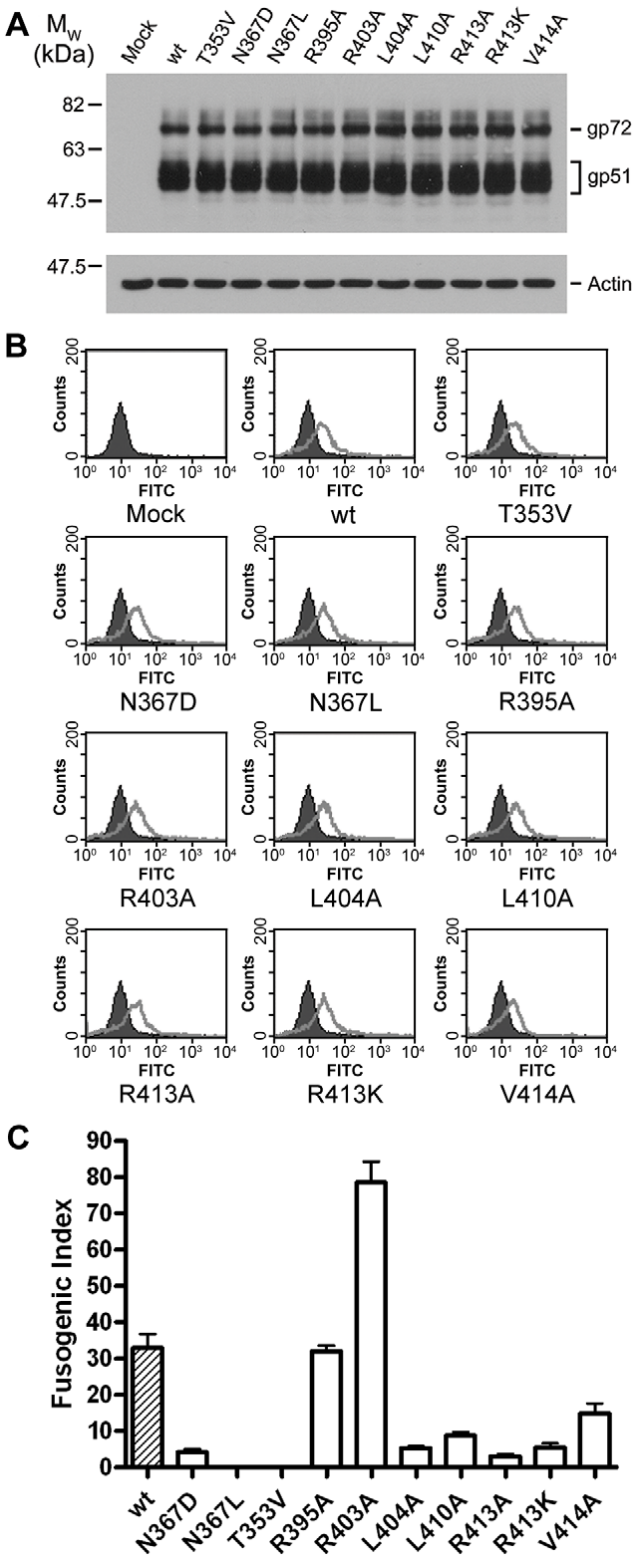
Expression and fusogenicity of mutant BLV envelopes. A panel of 10 mutants in BLV envelope was generated using site-directed mutagenesis of the pCMV-BLV*env*-RRE vector. The envelopes (or empty pCDNA3.1 for the mock sample) were transfected with pRSV-Rev into HeLa cells, and the cells were split into three aliquots, one sample was lysed for Western analysis, another stained and examined by flow cytometry, and the third used as effector cells in syncytium formation assays with non-transfected HeLa cells as target cells. (**A**) Western blot probed for SU (upper panel) and actin (lower panel). Upper band is the Env precursor, gp72, lower bands gp51. (**B**) Flow cytometry analysis using the same primary antibody as in (**A**), detected with FITC-labelled anti-mouse IgG. Solid histograms represent mock transfected cells, shown alone in the first panel, and open histograms represent the envelope transfected cells. (**C**) Fusogenicity of the mutant envelopes. Fusogenic index represents the number of nuclei in a low-power light microscope field present in syncytia as a percentage of the total nuclei in the field (Data represent the means from triplicate assays ±SD).

Guided by the crystal structure, a panel of mutants in BLV envelope were designed to perturb key features of the trimer-of-hairpins structure. A common difficulty with mutagenesis of viral envelopes, particularly the TM, is that at particular positions even conservative substitutions can dramatically impair the proteolytic processing and cell surface expression of envelope [27], [28]. Therefore, we compared each of the mutants with the parental “wild-type” envelope and confirmed appropriate expression, processing and cell surface presentation of the TM mutants. No significant differences in either the expression level of gp51 or the cleavage of the precursor protein, gp72, were observed (Fig. 2A). Moreover, flow cytometry analysis confirmed that in each case the mutant envelope was displayed on the cell surface at levels equivalent to wild type (Fig. 2B). Subsequently, the envelope-expressing cells were used as effector cells in syncytium formation assays, and the efficiency of each mutant envelope relative to wild-type in catalysing membrane fusion was calculated as the relative fusogenic index. The effect of each mutation on fusogenicity (Fig. 2C), and the mutant phenotypes are interpreted with reference to the crystal structure below.

### Solvent molecules are critical for stable assembly of the core coiled-coil

**Figure 3:**
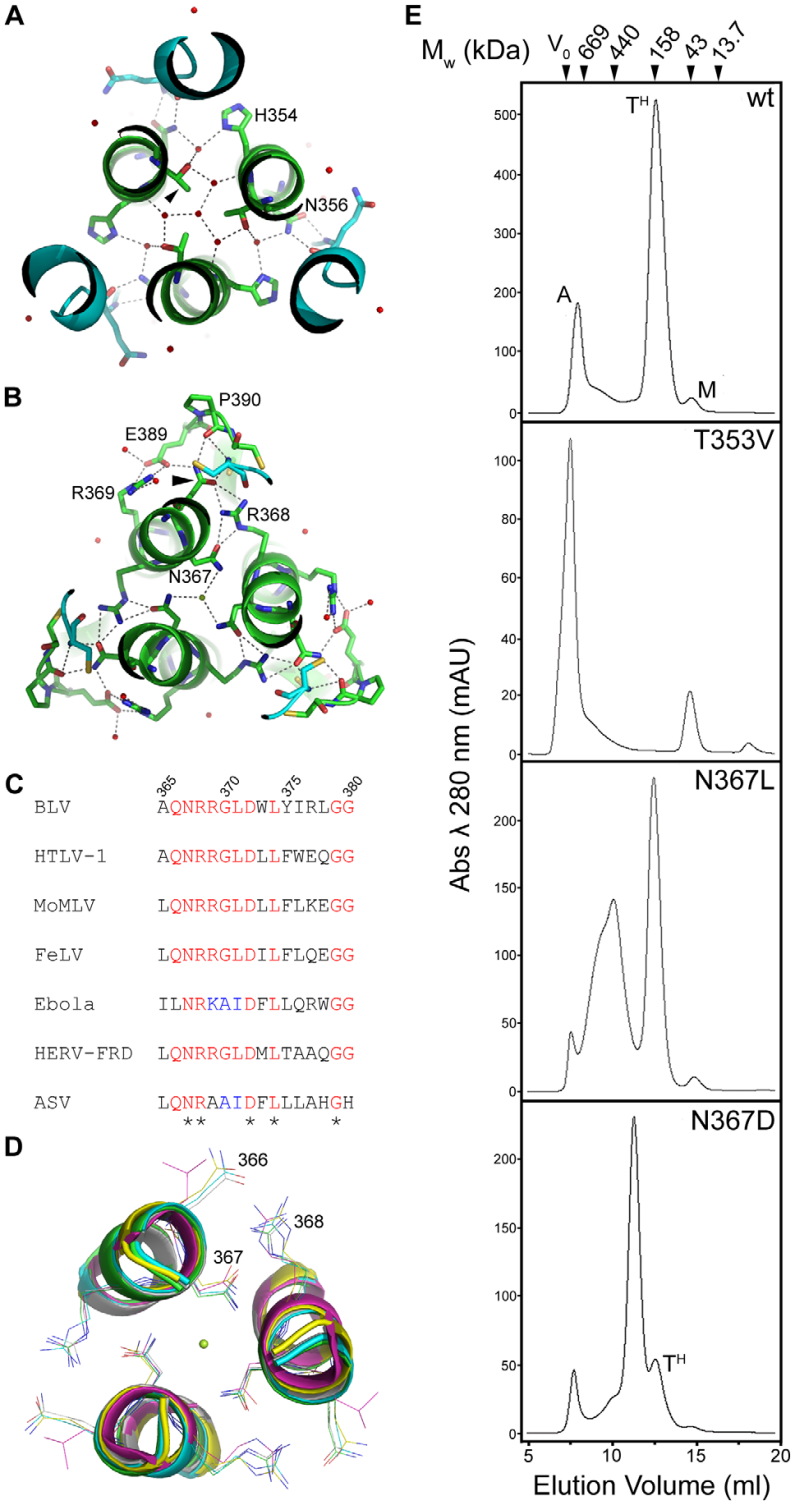
Role of solvent molecules in the trimerisation of the coiled coil. (**A**) and (**B**) Slice through the BLV trimer-of-hairpins showing the network of ordered small molecules in the coiled coil co-ordinated by Thr353 (**A**) and Asn367 (**B**). N-helices are shown as green ribbons with important residues shown as sticks and coloured according to atom type (green is carbon, red is oxygen and blue is nitrogen). LHRS are shown in turquoise. Red spheres represent ordered water molecules, the green sphere central in (**B**) represents a chloride ion. (**C**) Clustal W alignment of a small region of the N-helices from several viruses. Completely conserved residues are coloured yellow, semi-conserved in green. Note conservation of Asn367 (BLV co-ordinates), Arg368 and Asp372. (**D**) Structural superimposition of a slice through the coiled coils of BLV (green), HTLV-1 (yellow, PDB ID 1MG1), Syncytin (white, PDB ID 1Y4M), MoMLV (turquoise, PDB ID 1MOF) and Ebola (purple, PDB ID 2EBO). Alpha helices are shown as cartoons, conserved Asn367 (BLV co-ordinates), Arg368 and Asp372 are shown as sticks. The green sphere represents the chloride ion. (**E**) Gel filtration chromatography elution profiles of bacterially expressed MBP-hairpin constructs, with MBP fused to the wild type or mutant BLV TM ectodomain as indicated. Molecular weights indicated were determined using protein standards. “A” denotes aggregated material, “T^H^” denotes trimer-of-hairpins, “M” denotes monomeric protein.

The trimerisation of the N-helices appears to be facilitated by presentation of aliphatic residues to the interacting faces of the TM monomers, thereby forming a hydrophobic core down the axis of the central coiled coil. Notably, in the BLV trimer-of-hairpins there are two positions along this interface that harbour polar residues, Thr353 and Asn367 (Fig. 3A, B). Thr353 is located approximately half way up the N-helix, and it is oriented such that the methyl group of the side chain points toward the centre of the coiled coil and the hydroxyl group faces toward the neighbouring N-helix. In this position, Thr353 participates in a complex network of hydrogen bonding via several buried ordered water molecules. In particular, Thr353 makes a contact through one water molecule with the main-chain of Glu397 and through a separate water molecule to the side-chain of His354, one of the residues which forms the wall of the groove into which the LHR binds (Fig. 3A). In the context of envelope the T353V substitution, which replaces threonine with a non-polar residue of equivalent size, completely abrogates envelope-mediated membrane fusion (Fig. 2C). Moreover, the introduction of the T353V substitution into the pMBP-BLVhairpin vector yields a recombinant protein that completely fails to trimerise (Fig. 3E). Interestingly, the data demonstrate that substitutions at Thr353 do not affect expression, processing, or surface expression of native envelope but severely impair the fusogenic activity of envelope by preventing assembly of the trimer-of-hairpins. Our data therefore provides further evidence that the trimeric coiled coil likely forms during the fusion process.

### Interaction with chloride ions

A spherical density feature was observed on the central axis of the molecule; comparison of refined temperature factors with those of the surrounding protein atoms suggests an entity more electron-dense than water. Based on the chemical environment, the shape of the density and *B* factor comparisons we have modeled this feature as a chloride ion. This chloride ion is situated towards the chain reversal end of the coiled coil between a group of three asparagines, one from each monomer of TM (Asn367). These asparagine residues establish electrostatic interactions with the chloride ion by creating a slightly positively charged microenvironment between the N-helices. An alignment of envelope sequences from diverse viral groups (Fig. 3C) reveals that this asparagine is conserved. Moreover, comparison of the crystal structures for the fusion protein ectodomains of HTLV-1 [20], MoMLV [29], Ebola [24], [30], and Syncytin-1 [31], a protein derived from a human endogenous retrovirus that plays a critical role in the implantation of the trophoblast into the wall of the uterus [32], show that the ability to coordinate chloride is retained (Fig. 3D). To test the importance of the chloride interacting asparagines, we introduced amino acid substitutions of similar size and geometry. Introducing a leucine residue at position 367 of BLV TM renders envelope entirely non-fusogenic. The N367L substitution does not completely prevent trimerisation of the BLV coiled coil *in vitro* but it significantly destabilises the trimer and a large broad peak corresponding to higher order oligomers and aggregated material is observed by gel filtration chromatography (Fig. 3E). Interestingly, a substitution whereby Asn367 is replaced by an aspartic acid residue produces a decrease in fusogenicity of almost 90% (Fig. 2C). The N367D substitution replaces an amide with a carboxylate group in the centre of the coiled coil, which inverts the surface charge of the binding pocket, and therefore would not support interaction with a chloride. In contrast to N367L, the N367D mutation should maintain the hydrogen bonding in and around the chain-reversal region mediated by the carbonyl group that is common to both asparagine and aspartic acid, and therefore the disruption of fusion should be due primarily to the absence of the chloride ion, albeit we cannot exclude the possibility that the close proximity of negatively charged aspartate side chains impairs trimerisation. However, our biological assays demonstrate that the N367D substitution, unlike N367L, retains some membrane fusion activity. *In vitro*, rather than producing a range of higher order species, the introduction of the N367D mutation into pMBP-BLVhairpin produces a recombinant protein with a major peak at an elution volume consistent with the molecular weight of a tetramer rather than a trimer (Fig. 3E). Tetramerisation is unlikely to be the reason for the compromised fusogenic activity of the N367D-envelope; nonetheless, the results demonstrate the importance of the chloride-interacting asparagines to the stability of the trimeric coiled coil.

### The interaction of the LHR with the coiled coil

Along the length of the LHR multiple contacts are made with the coiled coil and such interactions are required for the activity of inhibitory LHR-mimetic peptides [14], [17], [21], [22]. Amino acid residues that are critical determinants of peptide potency map to the C-terminal region of the LHR [21], [22], but key residues and the manner in which they interact with the coiled coil are not resolved in the published HTLV-1 TM structure [20]. A detailed view of the BLV LHR bound to the coiled coil and a sequence comparison of the HTLV-1 and BLV LHRs are shown in Fig. 1C and D. Towards the N-terminus of the BLV LHR, conserved residues Leu394 and Ile396 dock into a hydrophobic pocket on the coiled coil, and an array of polar side chains at one side of the pocket interact with the LHR peptide backbone (Fig. 1C). We have previously demonstrated that interactions with the peptide backbone contribute to the activity of the BLV and HTLV-1 peptide inhibitors [17], [22]. By contrast, the side chain of Arg395 projects out into solvent suggesting that this residue is not essential for interaction of the LHR with the coiled coil or for trimer-of-hairpins formation (Fig. 1C). In keeping with this view, the R395A substitution has no significant effect on envelope fusogenicity (Fig. 2C) and therefore serves as a useful control for our analysis of envelope structure and function. As the N-helices twist around one another the groove between them accommodates the first conserved α-helical segment of the LHR. Within this α-helix, BLV residues Ile401 and Leu404 extend down and pack into the groove (Fig. 1C). Significantly, substitution of Leu404 with alanine results in an 80% loss of fusogenic activity relative to wild type (Fig. 2C), indicating that this interaction contributes substantially to the stability of the LHR/coiled coil interaction.

The first α-helix of the BLV LHR ends at Asp406, and between the end of this helix and the beginning of the next is a three-residue linker, comprised of Leu407, Gln408 and Pro409. Although Leu407 is positioned such that it faces the groove between N-helices, a ridge across this groove prevents Leu407 from docking particularly deeply (Fig. 1C). This Leucine is not conserved among leukaemia viruses. Instead, there is an Arginine residue at this position of the HTLV-1 LHR that makes contact with the coiled coil. Such differences may account, in part, for the specificity and significant differences in potency that are observed for the BLV and HTLV-1 peptide inhibitors [17]. The conserved proline (BLV Pro409) of the extended non-helical linker induces a sharp kink in the LHR thereby allowing a second α-helix to bind almost directly across the groove, at right angles to the rest of the LHR. Crucially, this second helix is not resolved in the published HTLV-1 TM structure [20]. At the point at which this change of direction occurs, a conserved leucine, Leu410, docks into the start of a deep hydrophobic pocket. Adjacent to Leu410 in this pocket and within the second LHR helix is Val414, which is also conserved in HTLV-1 (Fig. 1C, D). Substitution of either Leu410 or Val414 with alanine markedly impairs the fusogenic function of envelope (Fig. 2C). The substitution of L410A is somewhat more detrimental than substitution of Val414, with an activity loss of 75% and 65% respectively.

Embedded within the second helical element of the LHR is an arginine (Arg413) that participates in electrostatic interactions with the coiled coil (Fig. 1C). This residue is conserved in HTLV-1 and is critical to the inhibitory activity of the HTLV-1 LHR mimetic peptide P^cr^-400 [22]. Intriguingly, Arg413 projects back along the axis of the coiled coil and binds between Leu407 and Leu410, where it forms hydrogen bonds with Gln343 from the same N-helix and Asp342 from an adjacent N-helix and also donates a hydrogen bond, through the ε-nitrogen, to the main chain carbonyl of Glu408 of the LHR (Fig. 1C). The substitution of Arg413 with alanine dramatically disrupts the fusogenic activity of envelope and reduces envelope function by more than 90% relative to wild type (Fig. 2C). Moreover, Arg413 cannot be functionally replaced by lysine. The R413K mutant, while more fusogenic than the alanine substituted derivative, is still 85% less effective at catalysing membrane fusion than wild-type envelope (Fig. 2C). These data indicate that the electrostatic and hydrogen bonding interactions made by Arg413 are essential to the envelope-mediated membrane fusion process.

### Contrasting effects of two arginine residues on the stability of the trimer-of-hairpins

**Figure 4:**
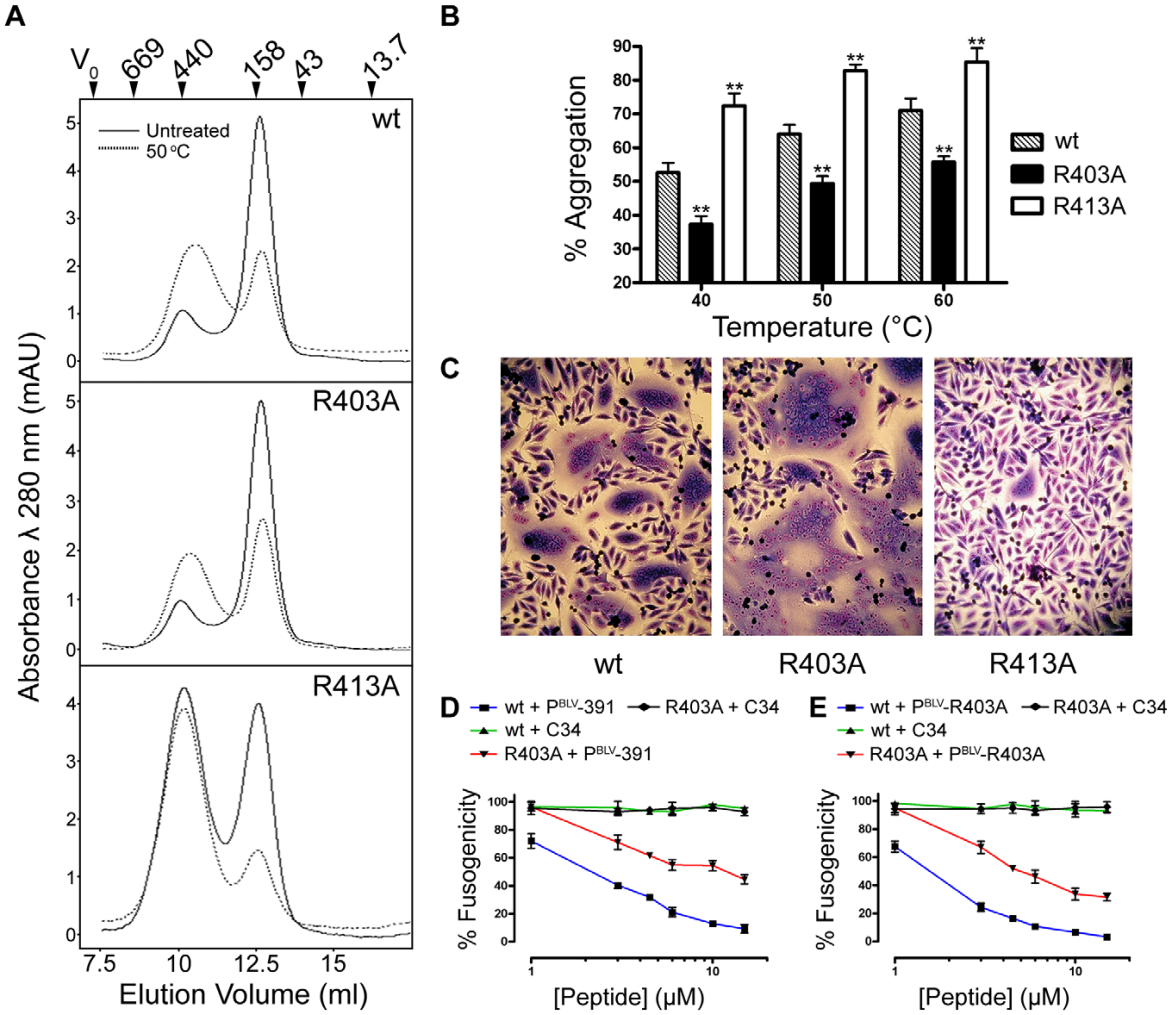
Contrasting effects of alanine substitutions at key arginine residues in the BLV coiled coil. R403A and R413A mutations were introduced into the MBP-BLV Hairpin vector, expressed and purified in bacteria and run over a size exclusion column to purify the trimeric species. The trimer-of-hairpins was then re-run over the column with or without heat treatment for 5 mins at 50°C. (**A**) Gel filtration elution profiles of non-heat treated (solid lines) or heat treated (dashed lines) mutant BLV MBP-hairpin proteins. Molecular weights indicated were determined using protein standards. (**B**) The area under the peaks corresponding to aggregated material from gel filtration experiments (example traces in (**A**)) was calculated as a percentage of the sum of the areas under both the aggregate peaks and the peaks corresponding to the trimeric species. Columns show the means from two experiments following heat treatment at each of the three temperatures shown. (**C**) Images of typical Giemsa-stained syncytia formed when cells transfected with wild-type, R403A or R413A BLV envelope expression constructs were used as effector cells in syncytium formation assays. (**D**) and (**E**) Membrane fusion induced by the R403A mutant envelope is less sensitive to inhibition by LHR-mimetic peptides. HeLa cells transfected with either wild-type or R403A mutant were used as effector cells in syncytium formation assays, in the presence of increasing amounts of BLV LHR mimetic peptide based on wild type LHR (P^BLV^-391, (**D**)), or, alternatively, incorporating the R403A substitution (P^BLV^-R403A, (**E**)) or control peptide (C34, HIV inhibitor). Data represent the mean ±SD from triplicate assays (syncytia from 5 low power light microscope fields were scored).

Based on homology modelling, we previously suggested that Arg403 of the LHR projects out from one side of the α-helix and is repelled electrostatically by N-helix residue Arg345 [17]. This prediction is substantiated by the crystal structure presented here (Fig. 1C). Moreover, the R403A substitution yields an envelope that produces extensive syncytia and is significantly more fusogenic than wild-type envelope (Fig. 2C and Fig. 4C). Thus, the substitutions R403A and R413A have very different effects on the fusogenicity of BLV envelope, increasing activity by over two-fold and almost completely abolishing activity respectively (Fig. 2C and 4C). Using a modified thermal aggregation assay [33], we assessed the relative effects of these two substitutions on the thermostability of the BLV trimer-of-hairpins. Control experiments revealed that after a five minute incubation at 40°C, on average 53% of the wild-type MBP-BLV-hairpin protein was present in high-order aggregates (Fig. 4A, B). However, when the same heat treatment was applied to the protein bearing the R403A mutation, we found that only 37% of protein aggregated. The difference to wild-type hairpin was significant (P≤0.005, *t*-test) (Fig. 4A, B). By contrast, the R413A substitution resulted in aggregation of over 70% of total protein, again a significant difference to wild type (P≤0.005, *t*-test) (Fig. 4A and B). Notably, even without heat treatment over 50% of the R413A protein was aggregated (Fig. 4A). Furthermore, these relative differences in the propensity of the recombinant proteins to aggregate were maintained following heat treatment at 50 and 60°C (Fig. 4B). Hence, the contrasting effects of the arginine substitutions on envelope fusogenicity are directly due to changes in stability of the post-fusion trimer-of-hairpins structure.

### Reduced sensitivity to peptide inhibitors

A prediction based on the increase in thermal stability of the R403A substituted trimer-of-hairpins is that such substitutions should confer reduced sensitivity to LHR-mimetic peptide inhibitors. To test this view we compared the activity of the P^BLV^-391 antagonist of envelope fusion [17] against wild type or R403A-substituted envelope. This peptide mimics residues Cys391 to Gln419 of BLV Env. In syncytium interference assays, P^BLV^-391 inhibited fusion catalysed by wild-type envelope with an IC_50_ of 3.17±0.09 µM (Fig. 4D). However, P^BLV^-391 only inhibited fusion catalysed by R403A envelope with an IC_50_ estimated to be >9 µM, and even at peptide concentrations up to 15 µM inhibition of syncytium formation was markedly damped indicating that both the potency and efficacy of the peptide was reduced against R403A substituted envelope (Fig. 4D). Moreover, though more active against native envelope, a reciprocally substituted peptide antagonist, P^BLV^-R403A, failed to exhibit full potency against the R403 envelope derivative; P^BLV^-R403A inhibited wild-type envelope catalysed membrane fusion with an IC_50_ of 1.3±0.1 µM, but inhibited the R403A envelope with an IC_50_ of >6 µM (Fig. 4E). In both experiments, C34 (a peptide mimetic of HIV-1 gp41 residues Gly627 to Leu661) was used as a negative control. Thus, improving the thermal stability of the trimer-of-hairpins form of a class-1 fusion protein significantly reduces the sensitivity of envelope to peptide inhibitors targeted to the coiled coil.

### A critical site for the binding of HTLV-1 small molecule fusion inhibitors

**Figure 5:**
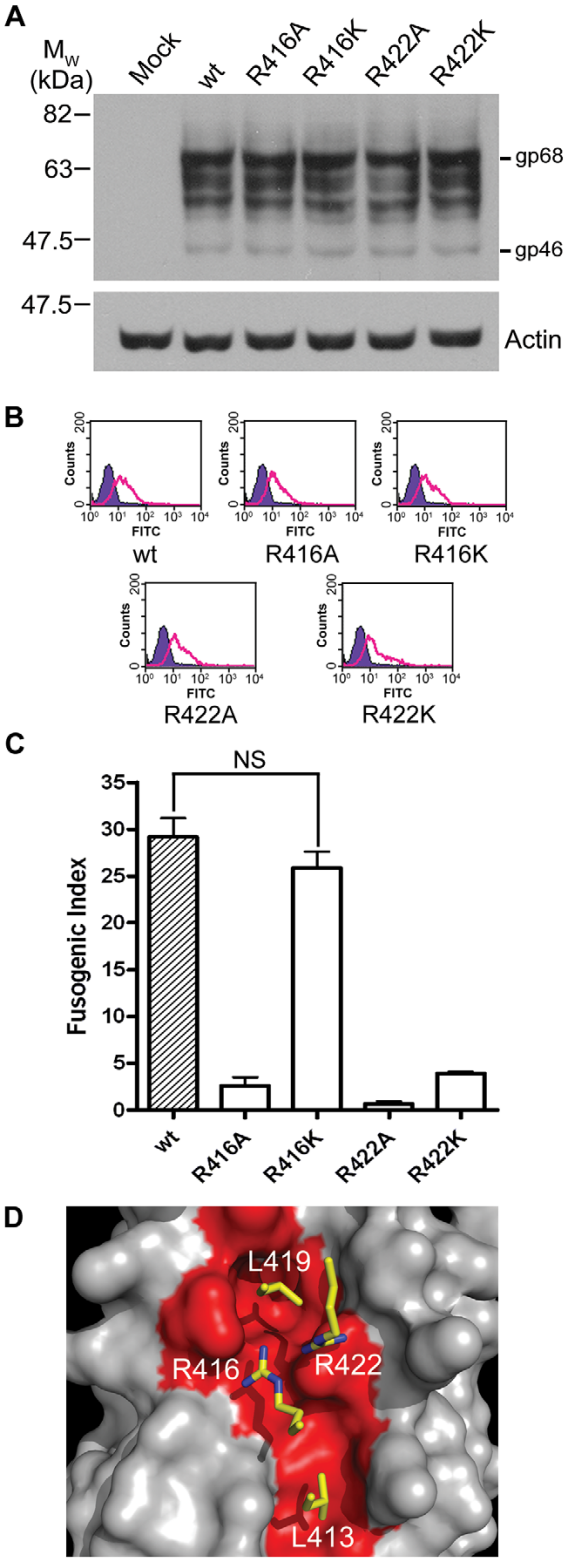
Basic residues in the C-terminal of the HTLV-1 LHR contact the coiled coil. The pCMV-HTLV*env*-RRE construct and derivatives encoding substitutions of Arg416 or Arg422 of HTLV-1 envelope were co-transfected with pRSV-Rev into HeLa cells, empty pCDNA3.1 transfected with pRSV-Rev was used as a negative control. Transfected cells were processed as in Fig. 3. (**A**) Western blot probed for HTLV SU (upper panel) or actin (lower panel); β-mercaptoethanol was used during sample preparation. (**B**) Flow cytometry of envelope expressing HeLa cells. Solid purple histogram illustrates the fluorescence profile of control cells, open green histograms show the fluorescence profile of cells transfected with the indicated envelope construct, all cells were probed for surface displayed envelope. (**C**) Fusogenicity of HTLV-1 C-terminal LHR arginine mutants. The fusogenic index was calculated as described (mean ±SD from triplicate assays). (**D**) A “hotspot” for binding of HTLV-1 fusion inhibitors based on the previously published HTLV-1 structure and modelled incorporating the data from this study. Four key residues (Leu413, Arg416, Leu419 and Arg422, shown as yellow sticks) interact with the HTLV-1 coiled coil (grey space-filling model, region interacting with LHR residues shown in red) within a compact binding site. The contact made by Arg422 is critical to the binding and functional activity of HTLV-1 peptide inhibitors.

The structure of the C-terminal segment of the BLV LHR and the accumulated data suggest a key role for the conserved arginine (Arg413) in the mechanism of envelope-mediated membrane fusion. Moreover, our recent data indicate that the equivalent arginine residue, Arg422, of the HTLV-1 LHR-mimetic peptide is critical to inhibitory activity [22]. Notably, for the HTLV-1 LHR-based inhibitor residues equivalent to Leu413, Arg416 and Leu419 are also of importance to the biological activity of the peptide [21], [22]. We therefore sought to establish whether or not the conserved arginine in the HTLV-1 LHR docks with the coiled coil in a similar manner to that of BLV and if this residue is important to membrane fusion mediated by the HTLV-1 TM. We generated a small panel of mutants in HTLV-1 envelope, substituting Arg416 of the LHR with alanine and lysine (R416A and R416K), and making similar substitutions for Arg422 (R422A and R422K). Using a monoclonal antibody recognising HTLV-1 SU, we confirmed by Western blotting and flow cytometry that all four mutant envelopes were expressed and post-translationally processed in a manner identical to wild type (Fig. 5A, B). In syncytium formation assays, the individual alanine substitutions of Arg416 and Arg422 resulted in envelopes that are 91% and 97% less fusogenic than wild-type envelope respectively (Fig. 5C). Significantly, the lysine substitution of Arg416 produced an envelope that was not significantly less fusogenic than wild type (P>0.05, *t*-test) (Fig. 5C). However, Arg422 could not be functionally replaced with lysine, the R422K mutation resulted in an 88% reduction in fusogenic activity. Taken together with the data from the BLV R413K mutant, this suggests that HTLV-1 R422 adopts a near-identical conformation to BLV R413 at the C-terminal segment of the LHR.

The likely conservation of position and orientation of HTLV-1 R422 in the trimer-of-hairpins allowed us to construct a model for the binding of the C-terminal segment of the HTLV-1 LHR to the coiled coil (Fig. 5D). The model suggests that R422 could bind to a negatively charged ridge that is orientated across the groove between N-helices and that lies between the binding pockets for Leu413 and Leu419 and that R422 docks adjacent to R416. As such, four critical interactions between LHR side chains and the coiled coil are contained within a binding hotspot of ∼16 Å x ∼8 Å and disruption of these interactions profoundly impairs envelope-mediated membrane fusion.

## Discussion

There is greater sequence divergence between BLV and HTLV-1 than, for example, between SIV and HIV-1 [34], [35], nonetheless the crystal structures of the BLV and HTLV-1 trimer-of-hairpins are similar. The incorporation of ordered water molecules and ions to facilitate trimerisation of the coiled coil and assembly of the trimer-of-hairpins is seen in several viral fusion proteins [20], [29], [30], [36]. The crystal structure of SIV gp41 implicates multiple water molecules in the folding of the trimer-of-hairpins [37]. However, the number of water molecules concentrated in one region of the BLV coiled coil is unusual. The interaction of Thr353 with an array of water molecules appears to play a critical role in assembly of the coiled coil, as substitution of T353 eliminates both *in vitro* trimerisation and *in vivo* fusogenicity. Notably, for HTLV-1 the threonine residue is replaced by an asparagine, which by virtue of its larger side chain directly hydrogen bonds with the adjacent N-helix. By contrast, Thr353 of BLV makes this contact through water-mediated hydrogen bonds. However, the contribution of polar interactions to coiled coil assembly is maintained at this location in HTLV-1 by interaction of a chloride ion on the symmetry axis of the coiled coil. Moreover, for both viruses three conserved asparagine residues located towards the membrane distal end of the coiled coil interact with a chloride ion. The properties of the N367L substitution and the failure of N367D to rescue envelope function indicate that the observed chloride-mediated interactions are essential for stable assembly of the coiled coil and for TM-mediated membrane fusion. Significantly, the asparagine (Asn367) and adjacent arginine (Arg368) residues of BLV are conserved between BLV, HTLV-1 and a number of disparate viral fusion proteins, and the ability of the asparagines to interact with a chloride ion is maintained. Critically, the chloride ion is present within the post-fusion conformation of Ebola GP2 (30), but is conspicuously absent in a pre-fusion structure resolved by Lee et al [38] wherein the conserved asparagines adopt a more open arrangement and the coiled coil is only partially trimerised. We suggest that Asn367, as a direct consequence of conformational changes in SU resultant from receptor binding, and in concert with the equivalent asparagines from neighbouring monomers, mediates electrostatic interactions with a chloride ion, which brings the N-helices together, thus driving the trimerisation of the coiled coil to yield the pre-hairpin intermediate in an event pivotal to successful fusion. In addition, our results indicate that trimerisation of the N-terminus of TM is not a requirement for Env maturation and surface display of trimeric pre-fusogenic envelope, offering a novel insight into the conformation of retroviral Env spikes prior to activation. Taken together, the data suggest that while the “knobs-into-holes” interactions of aliphatic residues at the centre of the coiled-coil regions of class 1 viral fusion proteins are important for trimerisation of the fusion-active coiled coil the polar layers among these hydrophobic interactions are critical. This information should be considered when designing trimeric immunogens based on the coiled coil of TM. Moreover, the structure in and around the chloride-interacting region has been conserved in diverse viral fusion proteins and may be susceptible to coiled coil destabilising drugs.

It is interesting to note that removal of a steric clash between R403 and the BLV coiled coil yields an envelope that is more fusogenic than wild type. For HTLV-1 an isoleucine, at a position equivalent to residue Arg403 in the BLV LHR, also appears to make a steric clash with the coiled coil and an alanine substitution of this residue in the context of an LHR-based peptide inhibitor yields a peptide with improved coiled coil-binding properties and increased inhibitory activity [21]. It is therefore clear that viral envelope has not evolved to optimise LHR binding to the coiled coil or to maximise fusogenicity. One plausible explanation is that membrane fusion needs to be kept in check. An overly fusogenic envelope may induce rampant membrane fusion and syncytium formation leading to cell death by apoptosis and thereby provide additional stimuli for a robust anti-viral immune response. Clearly, sub-optimal LHR/coiled-coil interactions have been maintained during viral evolution and suggest that the development of compounds that bind to the coiled coil with higher affinity than the LHR may be an achievable anti-viral strategy.

The most notable difference between the HTLV-1 and BLV TM structures is the resolution of a second helical motif within the LHR of the trimer-of-hairpins. Residues that form the start of the second α-helix are conserved in HTLV-1 and are critical to envelope-catalysed membrane fusion and to the inhibitory activity of LHR-mimetic peptides [21], [22]. Just three residues from the end of our structure is the first tryptophan of a conserved tryptophan-rich membrane-proximal region (MPR). The MPR is believed to interact with the opposing membranes during the fusion process and substitution of the aromatic residues severely compromises, but does not abolish, membrane fusion [39]. The conformation of the MPR has received considerable attention because broadly neutralising anti-HIV-1 antibodies bind epitopes contained within the MPR [40], [41]. It is likely that the HIV MPR adopts a helical structure when interacting with membranes and the coiled coil during fusion [40], [42]. Examination of the BLV envelope sequence and TM structure suggests that the second helical segment of the LHR is likely to continue and that in the fusion-activated state the tryptophan-rich MPR is also helical and inserted into the membrane at an oblique angle. For the leukaemia viruses, the conserved arginine (Arg413 in BLV), assists in defining the orientation of the MPR, as the interactions of Arg413 with the coiled coil and the LHR backbone propagate a sharp kink in the LHR towards the membrane-proximal end of the coiled coil. A lysine substitution of Arg413 retains only ∼10% of the fusogenic activity of wild-type envelope lending further support to this view.

**Figure 6:**
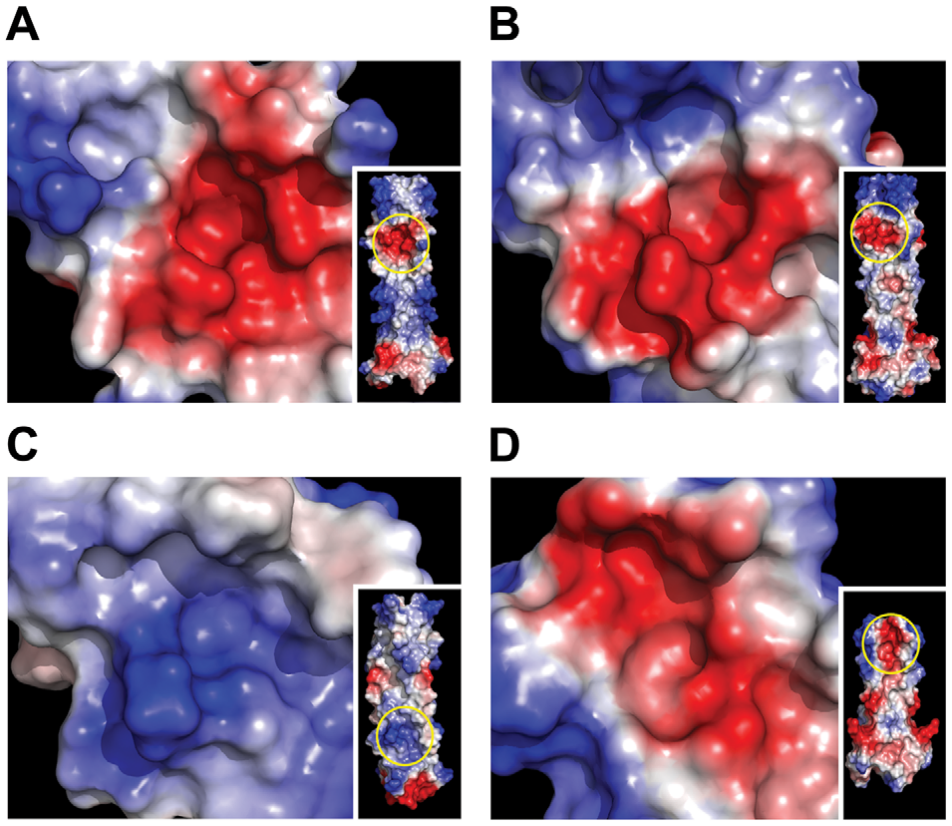
Electrostatic surface analysis of retroviral coiled-coils reveals the presence of key charged-surrounded pockets. The coiled coil surface of BLV (**A**), HTLV-1 (**B**), HIV (**C**) and MoMLV (**D**) is coloured according to electrostatic local protein contact potential using AMBER99 charges and radii, where blue represents net positive charge and red indicates net negative charge (+84.019 to -84.019 (kT/e)). Insets show the whole coiled coil surface, with the region magnified circled in yellow.

The BLV crystal structure and our analysis of HTLV-1 and BLV envelope-mediated membrane fusion reveal an important role for electrostatic interactions in binding of the LHR to the grooves of the coiled coil. For both BLV and HTLV-1 a conserved basic residue in the second helical segment of the LHR interacts with the charged rim of a deep pocket lined by non-polar residues. For HTLV-1 the contribution of charge is enhanced by the interaction of an additional basic residue (Arg416) with the opposite rim of the pocket. Thus, while the insertion of non-polar residues into hydrophobic pockets likely drives the docking of the LHR with the coiled coil, we suggest that it is the electrostatic attraction and hydrogen bonding of charged side chains around these hydrophobic interactions that serves to “cement” the LHR in place and thereby establish the precise geometry and affinity of this interaction. In keeping with this view, substitution of these basic residues ablates envelope-mediated membrane fusion and dramatically impairs the inhibitory and coiled-coil-binding properties of LHR-based peptides [22]. Moreover, only one of these important basic residues is conserved in BLV compared to HTLV-1 and it is notable that the synthetic BLV LHR-mimetic is approximately 10-fold less potent than the corresponding inhibitor of HTLV-1 [17]. Examination of the electrostatic surfaces of BLV, HTLV, HIV and MoMLV coiled coils reveals, in each case, the presence of a pocket surrounded by charged residues, though the polarity of the charge and positioning along the groove is variable (Fig. 6). The importance of the pocket and surrounding charge to the assembly of the trimer-of-hairpins and the potency of C-helix-based mimetic peptides has been demonstrated for HIV [43], [44], and taken with our findings for BLV and HTLV-1 suggest that such charged regions are critical to retroviral envelope-mediated membrane fusion in general. We suggest that structure-assisted design of small molecules to target these charge-surrounded pockets is a viable objective for anti-retroviral therapy.

We expect that the data produced in this study will be pivotal to the development of more drug-like inhibitors of HTLV-1 envelope catalysed membrane fusion, and thereby provide therapeutic antiviral agents for adult T-cell leukaemia and HTLV-associated myelopathy/tropical spastic paraparesis, diseases for which there are currently no effective treatments [45], [46].

## Methods

### Plasmid

The plasmids pCMV-BLV*env-*RRE and pRSV-Rev have been described [17], [47]. To construct pCMV-HTLV*env*-RRE the BLV *env* sequences in pCMV-BLVenv-RRE were replaced by a HTLV-1 *env* coding region amplified by PCR from pHTE-1 [48]. To construct the plasmid pMAL-gp30hairpin (MBP-BLV-hairpin), a modified fragment of the MBP open reading frame from pMAL-c2 (New England Biolabs) with a 5′ BglII site and a 3′ PvuII site replacing a SacI site was PCR amplified. A fragment of BLV *env* encoding amino acid residues 326 to 418 was PCR amplified with a 5′ PvuII site and a 3′ PstI site. The PCR fragments were ligated into a pMAL-c2 backbone digested with BglII and PstI. The Quikchange mutagenesis kit (Stratagene) was used to introduce point mutations into pCMV-BLV*env-*RRE and pMAL-gp30hairpin following the manufacturer's instructions.

### Expression and purification of the recombinant trimer-of-hairpins

Expression of the MBP fusion protein was carried out as previously described [14], [49]. Briefly, *Escherichia coli* BL21(DE3) cells transformed with the MBP-BLV-hairpin vector were grown at 37°C with vigorous shaking in LB supplemented with 10 mM glucose and 100 µgml^−1^ ampicillin until an optical density at 600 nm of ∼0.6 was reached. Protein expression was induced with Isopropylthio-β-D-galactoside (IPTG) at a final concentration of 0.5 mM for 4 h at 37°C. Cells were harvested by centrifugation and the pellet was resuspended in column buffer (20 mM Tris-HCl [pH 7.5], 200 mM NaCl, 1 mM EDTA) supplemented with protease inhibitors (1 mM phenylmethylsulfonyl fluoride and 1 µgml^−1^ aprotinin) and frozen O/N at −20°C. The cell suspension was thawed and the cells lysed by sonication. Cell debris was pelleted by centrifugation at 9,000×*g* for 30 min. The crude lysate was diluted 1∶5 in column buffer and loaded onto an amylose column pre-equilibrated with column buffer. The column was then washed with 15 column volumes of column buffer, and the bound protein was eluted with column buffer supplemented with 10 mM maltose.

### Protein crystallization and structure determination

The trimeric recombinant MBP-BLV-hairpin protein was purified by Superdex 75 gel filtration, and the required fractions pooled and concentrated to 12 mg ml^−1^. Tris [2-carboxyethyl]phosphine hydrochloride (TCEP-HCl) was then added to a final concentration of 5 mM. Protein concentration was estimated by absorbance measured at 280 nm. Crystallization conditions were identified using the sitting drop vapour diffusion technique, with crystal screens from Hampton Research and Emerald Biostructures. Optimal crystallization was achieved by streak seeding with a reservoir solution composed of 24.5% (v/v) isopropanol, 13.5% (v/v) PEG-4000 and 0.1 M sodium citrate pH 5.6, and optimal diffraction was achieved using a cryoprotectant composed of mother liquor supplemented with 20% (v/v) glycerol. Diffraction data was collected at beamline BM14 at the European Synchrotron Radiation Facility (ESRF) in Grenoble. The structure was solved by molecular replacement using a truncated model of MBP-HTLV Hairpin (PDB ID 1MG1). Refinement proceeded through cycles of model building using Coot and O [50], [51] and refinement using REFMAC5 [52]. Data and refinement statistics are given in Table 1. The atomic coordinates and structure factors (code 2XZ3) have been deposited in the Protein Data Bank, Research Collaboratory for Structural Bioinformatics, Rutgers University, New Brunswick, NJ (http://www.rcsb.org/).

**Table 1:** Data collection and refinement statistics for MBP-BLV-Hairpin.

Crystallographic Statistics	
Space group	R3
Cell dimensions	a = b = 107.60 Å, c = 119.17 Å, α = β = 90°; γ = 120°
Resolution range (Å)	27.00 - 1.95
Total measurements	131833
Unique reflections	37148
Completeness	0.990 (0.904)
*R_merge_*	0.045
Redundancy	3.5 (2.9)
*I*/*σ(I)*	23.9 (2.1)
*Wilson B (*Å^2^)	34.3
*Matthew's coefficient (*Å^3^)	2.63
Solvent content	0.53
Number of protein residues	459
Number of solvent molecules	285
Number of other residues	1
R_work; R_free	0.1890; 0.2368
Average *B*-factor (Å^2^)	
Overall	36.4
Protein Backbone	34.9
Protein side chains	37.1
Solvent	42.5
Other	27.1
RMSD Bonds (Å)	0.016
RMSD Angles (°)	1.65
Ramachandran plot statistics [2]	91.8%
Most favoured region	8.2%
Additional allowed region	N/A

Values in parentheses pertain to the highest resolution shell of 0.05 Å.

[2] as determined by PROCHEK.

### Peptides

Peptides were synthesised using standard solid-phase Fmoc chemistry and unless stated otherwise have acetylated N-termini and amidated C-termini. The peptides were purified by reverse-phase high-pressure liquid chromatography and verified for purity by MALDI-TOF mass spectrometry. All peptides were dissolved in dimethyl sulfoxide (DMSO), the concentration of peptide stock solutions was confirmed by absorbance at 280 nm in 6M guanidine hydrochloride and peptides were used at the final concentrations indicated.

### Cells and syncytium formation assays

HeLa cells were maintained in Dulbecco's modified Eagle medium supplemented with 10% foetal bovine serum (FBS). For syncytium formation assays, HeLa cells were transfected with equal quantities of pCMV-BLV*env*-RRE (or empty pcDNA 3.1 for mock samples) and pRSV-Rev using the GeneJuice transfection reagent (Novagen). After 24 h, 3×10^5^ transfected cells were added to 7.0×10^5^ non-transfected HeLa target cells in 6-well dishes (Nunc), and cocultured for 16 hrs, in the presence of peptides where appropriate. The cells were washed with phosphate-buffered saline pH 7.4 (PBS), fixed using 3% paraformaldehyde in PBS, and stained with Giemsa. Assays were performed in triplicate. To calculate fusogenic indices, the fraction of nuclei contained within syncytia in a 20x light microscope field was expressed as a percentage of the total number of nuclei within the field.

### Western blotting

Western blotting was carried out using standard methods; β-mercaptoethanol was used in sample preparation. BLV and HTLV-1 envelope was detected using supernatant from murine hybridoma cell lines expressing monoclonal antibodies raised against recombinant antigen derived from BLV or HTLV-1 envelope, and anti-mouse horseradish peroxidase conjugated secondary antibody at a dilution of 1∶10,000 in PBS containing 5% (w/v) marvel and 0.025% Triton X-100.

### Flow cytometry

To detect surface-expressed protein, cells transfected with the appropriate envelope expression vector were detached from culture flasks using PBS +2 mM EDTA, and washed twice with PBS. 5.0×10^5^ cells were incubated with agitation for 1 hr at room temperature in 1 ml DMEM +10% FBS containing 15 µgml^−1^ immunoglobulin purified from the BLV anti-Env antibody-expressing hybridoma supernatant, or 150 µl supernatant from the HTLV anti-Env antibody expressing hybridoma. Cells were washed twice with PBS and incubated in the dark at room temperature for 45 mins in DMEM containing anti-mouse FITC (Sigma) at 1/1,000 dilution. Cells were washed once with PBS and once with PBS +0.1% sodium azide before fixing with PBS +0.5% paraformaldehyde. Bound fluorescence was detected using a FACScan flow cytometer (Becton Dickinson).

### Gel filtration chromatography

The oligomerisation states of MBP-BLV-hairpin and mutant derivatives were examined by gel filtration using a Superdex 200 column equilibrated with MBP elution buffer. Fractions containing the trimeric species were retained. To assess the thermostability of the trimers, samples from these fractions were re-run over the column either without heat treatment or with heat treatment at the temperature specified for 5 min, followed by cooling for 2 min on ice. The areas under the peaks observed were calculated, and the area of the peak corresponding to the aggregate was expressed as a percentage of the total area under both this peak and the peak corresponding to the trimer.
